# Dry Eye and Corneal Langerhans Cells in Systemic Lupus Erythematosus

**DOI:** 10.1155/2015/543835

**Published:** 2015-03-29

**Authors:** Miklós D. Resch, László Marsovszky, János Németh, Márta Bocskai, László Kovács, Attila Balog

**Affiliations:** ^1^Department of Ophthalmology, Semmelweis University, Mária Utca 39, Budapest 1085, Hungary; ^2^Department of Rheumatology, Albert Szent-Györgyi Health Center, University of Szeged, Kálvária Sugárút 57, Szeged 6725, Hungary

## Abstract

*Purpose*. Investigation of dry eye and corneal Langerhans cells (LCs) in systemic lupus erythematosus (SLE). *Methods*. Prospective consecutive case series of 27 SLE patients and 27 control subjects. Dry eye was evaluated by lid-parallel conjunctival folds (LIPCOF), Schirmer test, tear break-up time (TBUT), and ocular surface disease index (OSDI) questionnaire. In vivo investigation of corneal LCs density and morphology (LCM) was performed with confocal corneal microscopy (Heidelberg Retina Tomograph with Rostock Cornea Module). *Results*. Tear production and stability were pathological in SLE subjects compared to control (Schirmer: 8.45 ± 9.82 mm/5 min versus 11.67 ± 3.21 mm/5 min; TBUT: 6.86 ± 3.53 s versus 11.09 ± 3.37 s). OSDI was significantly greater in SLE patients (25.95 ± 17.92) than in controls (11.06 ± 7.18). Central LC density was greater in SLE patients (43.08 ± 48.67 cell/mm^2^) than in controls (20.57 ± 21.04 cell/mm^2^). There was no difference in the peripheral LC density (124.78 ± 165.39 versus 78.00 ± 39.51 cell/mm^2^). LCM was higher in SLE patients in the centre (1.43 ± 0.79) and in the periphery (2.89 ± 0.42) compared to controls (centre: 1.00 ± 0.69, periphery: 2.35 ± 0.54). *Conclusions*. Significant changes in dry eye parameters and marked increase of central LCs could be demonstrated in SLE patients. SLE alters not only the LC density but also the morphology, modifies corneal homeostasis, and might contribute to the development of dry eye.

## 1. Introduction

Systemic lupus erythematosus (SLE) is a chronic systemic autoimmune disease of unknown etiology. It can manifest in the inflammation of various organs including skin, heart, joints, blood vessels, liver, kidneys, and nonetheless ocular tissues such as cornea [1, 2]. SLE is characterized by a hypersensitive systemic inflammatory reaction in a wide spectrum of tissues, and hence it may cause a wide range of clinical signs and symptoms [3]. Although several scoring systems have been validated to measure disease activity in SLE [4, 5], no objective laboratory or clinical marker has been identified that can reliably be used for the detection of ongoing inflammation in correlation with clinical symptoms.

The cornea is endowed with heterogeneous populations of immune cells including antigen-presenting dendritic cells [6, 7]. Amongst them corneal Langerhans cells (LCs) play a major role in corneal immune responses by activating T-cells and participating in the maintenance of corneal homeostasis. LCs are localized exclusively in the corneal epithelium and reside mainly in the peripheral cornea under nonpathological circumstances [8–12]. In vivo confocal corneal microscopy can provide an in vivo method to detect LCs in the corneal epithelium [13–15]. Pathological conditions, various forms of injuries [16], or minor stimuli to the cornea (such as contact lens wear [17] or chronic use of antiglaucoma eye drops [18]) may induce LCs to undergo maturation with the formation of dendrite-like processes [17, 18]. During this maturation LCs migrate from the periphery to the area concerned with further LC influx toward the central cornea [17].

Our team has recently demonstrated the presence of activated LCs in the central cornea of rheumatoid arthritis (RA) and ankylosing spondylitis (AS) patients [19, 20]. This drove our attention to further investigate corneal LCs and the dry eye related parameters in another systemic inflammatory disease, that is, SLE. Our hypothesis was that SLE alters the ocular surface homeostasis and increases the LC density and activation proportional to the severity of the general inflammation and disease activity of SLE.

## 2. Methods

### 2.1. Patients

This study was a prospective controlled consecutive case series of 27 SLE patients and 27 age- and gender-matched control subjects. The study has been performed in accordance with the ethical standards laid down in the Declaration of Helsinki. Written informed consent was obtained from all subjects, and our study was reviewed and approved by an independent ethics committee of the institution. The inclusion criteria were the presence of SLE diagnosed and classified according to the 1997 updated American College of Rheumatology (ACR) criteria [21] and a negative ocular medical history. The exclusion criteria were any known ophthalmic disease, contact lens wear, previous injury of the cornea, uveitis, and cataract. SLE patients with secondary Sjögren's syndrome diagnosed according to the 2002 American-European consensus criteria for Sjögren's syndrome were also excluded from the study [4], since it has been described that Sjögren related hypolacrimation resulted in severe dry eye, which itself results in significant changes in LC density and morphology. Our study was focused on patients with controlled SLE without known ocular symptoms.

### 2.2. SLE Disease Activity Evaluation

The SLE patients had various disease durations and activities. Disease onset was defined as the time when the patients fulfilled the criteria for SLE. Disease duration represented the period from the disease onset to the time of this study. Systemic lupus erythematosus disease activity index (SLEDAI) [4, 5, 22] score was calculated to determine disease activity for SLE on the day of ophthalmic assessments. Patients with an SLEDAI score of 0 were considered to be in remission, an SLEDAI score between 1 and 8 was regarded as moderate disease activity, and an SLEDAI score above 8 was regarded as high disease activity.

In addition, further descriptive laboratory parameters of general immune status, C-reactive protein (CRP) and erythrocyte sedimentation rate (ESR), were documented, of which we used CRP for further analysis in this study.

### 2.3. Evaluation of Dry Eye Parameters

The protocols for these procedures have been described in great detail in our previous works [19, 20]. Lid-parallel conjunctival folds (LIPCOF) were determined at the temporal aspect of lower eyelid margin according to the protocol by Pult et al. [23]. Tear production was measured by Schirmer test strip (Haag-Streit, ref. 4701001, UK) without anaesthesia. Evaluation of tear break-up time (TBUT) was carried out one minute after instillation of fluorescein dye into the lower conjunctival sac. The mean value of three consecutive measurements was interpreted as TBUT. The subjective discomfort was evaluated with the ocular surface disease index (OSDI) questionnaire [24].

All examinations were carried out on the right eye only and in the same room under constant conditions.

### 2.4. In Vivo Confocal Corneal Microscopy

In vivo confocal corneal microscopy was performed to quantify density and determine morphology of the LCs. The Heidelberg Retina Tomograph with Rostock Cornea Module (HRT II RCM) (Heidelberg Engineering Inc., Heidelberg, Germany) equipped with an inbuilt software of Heidelberg Eye Explorer (version 1.5.10.0.) was used. The ocular surface was anaesthetized with topical anaesthetic eye drops (Oxybuprocaine-Humacain 0.4%, Human Pharmaceuticals, Gödöllő, Hungary). Fixation of the patient's eye was maintained with a target mobile red light for the contralateral eye. A disposable plastic cap (TomoCap; Heidelberg) was used to keep the distance from the corneal surface to the microscope head stable. Carbomer gel (Vidisic; Dr. Mann Pharma, Berlin, Germany) was used as a coupling medium.

LC densities were examined both in the centre and in the corneal periphery at 6 o'clock according to the established examination scheme of Zhivov et al. [13, 17]. Thirty images were taken of the right eye and the five best-focused images were considered for the analysis in a masked fashion as described previously (independent evaluator masked for medical history or ophthalmological status of the patient) [19, 20]. After identification of LCs (bright, mostly oval or elongated particles with a diameter of up to 15 *μ*m) and selection of the region of interest, cells were manually marked, and the software automatically calculated cell density (cell number/mm^2^). LCs morphology (LCM) was evaluated on a 0–3 scale according to the size of the dendrites compared to the largest diameter of cell body. The length of all dendrites of all LCs in each image was measured with the inbuilt caliper. A score 0 described the condition when cornea was devoid of LCs. A score 1 was given when cells lacked processes. A score 2 (small processes) was given if the length of the processes did not exceed the longest diameter of the cell body. A score 3 (long processes) was given if the processes were longer than the largest diameter of the cell body. The average of LCM was calculated in each of the figures selected and was used to describe the maturation of the LCs at both regions of the cornea.

### 2.5. Statistical Analysis

Statistical analysis was performed using STATISTICA version 11.0 (StatSoft, Tulsa, OK, USA) software. For the comparison of control and SLE groups Mann-Whitney *U* test was performed. Central and peripheral LC density and morphology values were compared applying the Wilcoxon test. Subgroup analysis was performed with Kruskal-Wallis and Mann-Whitney tests. Fisher exact test was applied to compare groups concerning presence or lack of LC in the cornea. In all tests *P* < 0.05 was considered significant.

## 3. Results

The mean age of SLE patients (*N* = 27, 42.8 ± 11.4 years) was not different from the age of control subjects (*N* = 27, 40.4 ± 19.3 years, *P* < 0,01, Mann-Whitney *U* test). All examinations could be performed on every patient, and no further exclusion was necessary. Male/female ratio was identical in SLE (2/25) and control (6/21) groups (*P* = 0.18, Fisher exact test). Disease duration was 10.5 ± 8.9 (min. 0.5, max. 36) years. Disease activity was generally mild as the mean SLEDAI score was 3.21 ± 4.34 (0–19). SLEDAI score was zero in eight of the twenty-seven patients and only three patients exceeded the SLEDAI score of 8 which is indicative of a high disease activity. CRP (6.4 ± 8.3 mg/L) and ESR (27.3 ± 19.1 mm/h) values revealed a controlled systemic inflammatory status. Majority of patients (96%) were on systemic glucocorticosteroid and chloroquine (85%) therapy; 40% received azathioprine and 22% methotrexate.

### 3.1. Dry Eye Parameters

Table 1 summarizes our dry eye results. Of note, significant differences were detected in three out of four dry eye related parameters between SLE and control groups: Schirmer test and TBUT values were lower in SLE patients than in controls (Mann-Whitney test, *P* < 0.05), and OSDI scores were greater in SLE patients than in control patients (Mann-Whitney test, *P* < 0.01) (Table 1).

Subgroup analysis showed that, in cases of complete remission (SLEDAI = 0), only TBUT was lower in healthy subjects (Mann-Whitney test, *P* < 0.05), but LIPCOF, Schirmer, and OSDI were not different from control. In mild disease activity (SLEDAI 1–8), TBUT and OSDI, while in high systemic activity of SLE (SLEDAI > 8), TBUT, OSDI and Schirmer showed significant difference in comparison to control. LIPCOF was not affected by SLEDAI. In patients with SLEDAI > 8, extremely low tear production was revealed (Schirmer 2.16 ± 0.76 mm/5 min), and tear film stability was deteriorated (TBUT 3.66 ± 1.52 sec). No significant difference in LIPCOF could be demonstrated in any subgroup. In patients with SLEDAI > 8, extremely low tear production was revealed (Schirmer 2.16 ± 0.76 mm/5 min), and tear film stability was deteriorated (TBUT 3.66 ± 1.52 sec).

### 3.2. In Vivo Confocal Corneal Microscopy

Table 2 demonstrates our confocal microscopy results with regard to the different groups. LCs were present in the central and peripheral cornea of all SLE patients, while in controls only 8/27 expressed LC in the central cornea (*P* < 0.01, Fisher exact test).

**Figure 1:**
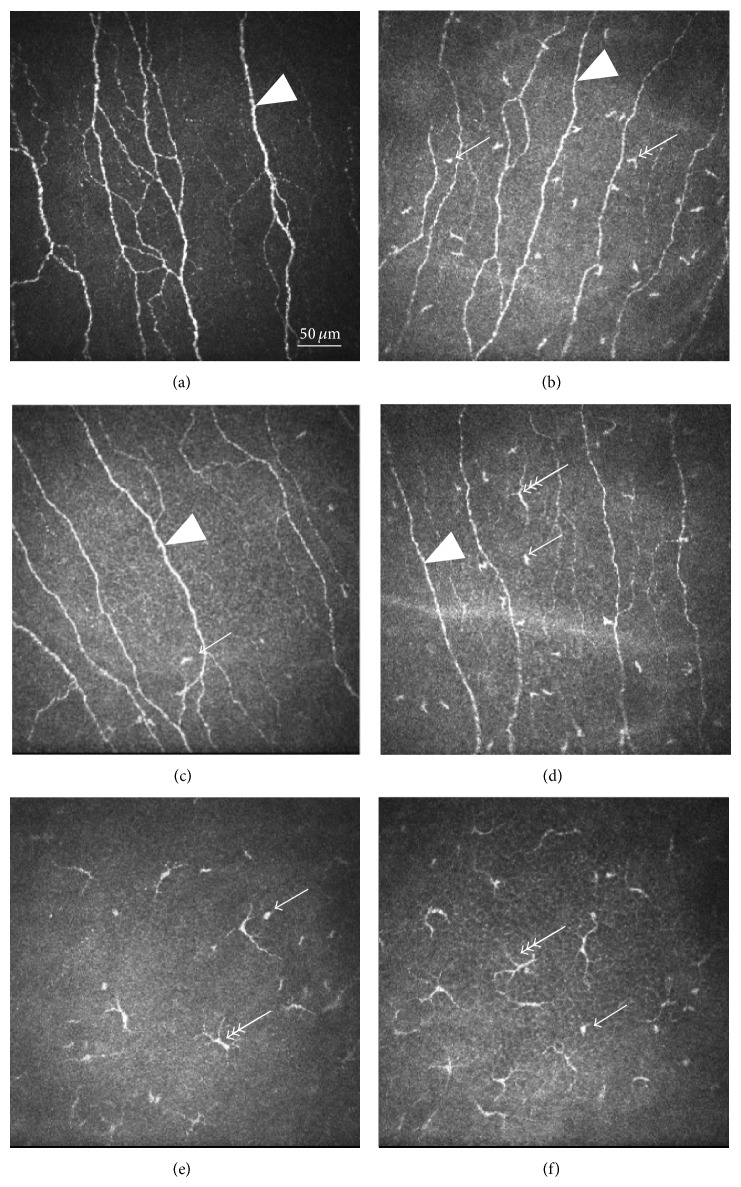
In vivo confocal microscopic images of corneal dendritic (Langerhans) cells (LC). Size of all images is 400 *μ*m × 400 *μ*m. Bar indicates 50 *μ*m. Simple arrow indicates LC without dendrite (LCM score = 1, probably immature), double arrow corresponds with LCM score = 2 (dendrite not longer than cell body), and triple arrow demonstrates LC with long dendrites (LCM score = 3, dendrite longer than cell body, sign of activation). Arrowhead points to subepithelial nerve plexus. (a) Image of the central cornea of a healthy volunteer. Note the normal subepithelial nerves. No LC is visible in the centre of the cornea (LCM score = 0) (depth of image from surface 43 *μ*m). (b) Central cornea of a patient with SLE. Density of LCs is larger than that in normal corneas. Most of the LCs are of LCM score 1, but some are of LCM score 2 (depth of image from surface 48 *μ*m). (c) Peripheral cornea of the same control eye as in Figure 1(a). Note that some LCs can be observed (only of LCM score 1). (d) Peripheral corneal image of the same patient as in Figure 1(b). Mixed population of LCs of LCM scores 1 and 3 can be seen (depth of image from surface 46 *μ*m). (e) Image taken anteriorly from subepithelial nerve fibres (37 *μ*m from surface) of the central cornea of an SLE patient. A large number of LCs are of LCM score 3 and some are of LCM score 1. (f) Peripheral cornea of the same patient as in Figure 1(e) (36 *μ*m from the surface). Density of LCs (predominantly LCM Score 3) is larger than that in the centre of the same cornea.

First we compared SLE patients to control subjects (Mann-Whitney test). Central LC density was greater in SLE patients than in controls (*P* < 0.05, Figures 1(a)-1(b)), while there was no significant difference in the peripheral LC density between SLE patients and controls (*P* = 0.213, Figures 1(c)-1(d)). LCs resided predominantly at the level of subbasal nerves; however in SLE patients we could identify LCs even anterior to the nerve fibres either in the centre and/or in the periphery (Figures 1(e)-1(f)). No LC could be detected in stromal layers.

Examination of LC morphology revealed that not only the density of LC but also the proportion of LCs with longer protrusions was increased in SLE, which is firmly believed to represent maturation and potential activity (Table 2, Figure 1). Indeed central LCM was higher in SLE patients compared to controls (score 1–3) (*P* < 0.05), where we have seen mostly immature dendritic cells (absence of processes and protrusions, score 0-1) in controls.

**Figure 2:**
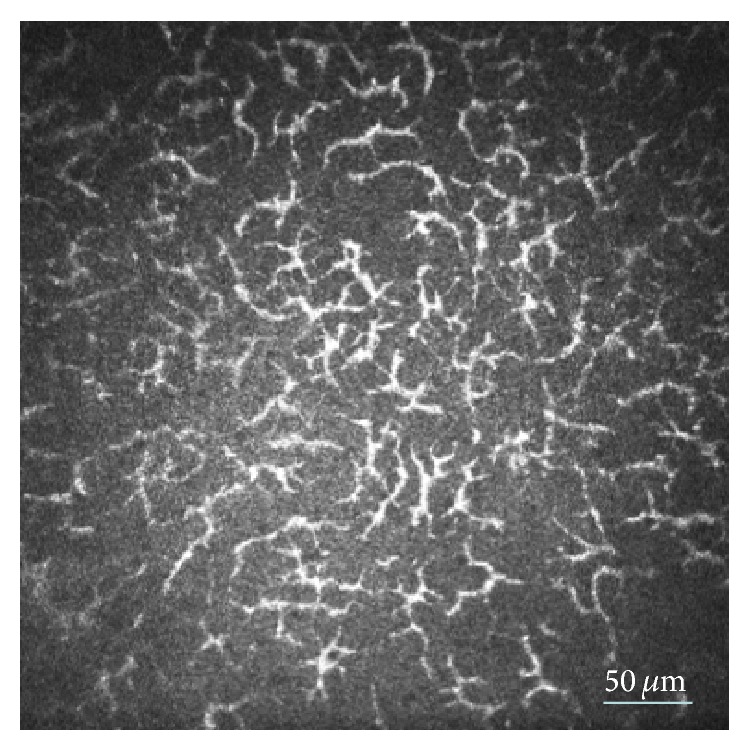
In vivo confocal microscopic image of corneal dendritic (Langerhans) cells (LC). Original size of the image is 400 *μ*m × 400 *μ*m. Bar indicates 50 *μ*m. Network of activated LCs with dendrites in an extreme large density in the central cornea of an SLE patient (depth of image from surface 41 *μ*m).

To gain a better understanding of the different factors which might operate on the LCs we performed subgroup analysis in the SLE group according to the following variables: SLEDAI, CRP, and Schirmer test. In patients with high systemic activity of SLE (SLEDAI > 8), central LC density was 46.10 ± 76.99 cell/mm^2^ and peripheral LC density was 133.20 ± 73.44 cell/mm^2^. The mean peripheral LCM in SLEDAI > 8 was 3.0, which means that all LCs displayed elongated dendrites, revealing a high level of activity. In some cases these activated LCs formed a network (Figure 2). We found that the central LC density was greater in SLE patients when the CRP was above 5 mg/L, compared to control subjects, but LC number was comparable to controls in SLE patients with lower inflammatory reactions (CRP < 5 mg/L). No significant difference was detected between the CRP determined subgroups. Subgroup analysis of SLE patients with compromised tear production (dry eye: Schirmer < 10 mm/5 min) and nondry eye (Schirmer ≥ 10 mm/5 min) revealed that all LC parameters (central and peripheral density and central and peripheral morphology) were pathological in SLE patients with lower tear production in comparison to controls. No difference was found between the subgroup of SLE patients with normal tear production and control subjects in the LC density and central LCM values; only peripheral LCM was higher in SLE patients with normal tear production than in control subjects.

**Figure 3:**
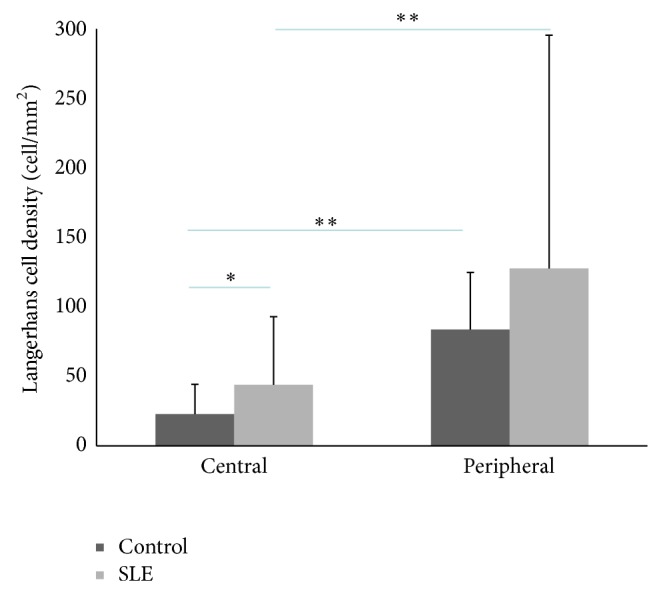
Diagram of the corneal Langerhans cell density in the central and peripheral areas. Centrally significantly greater value was shown in patients with SLE than in control group (^∗^ *P* < 0.05, Mann-Whitney test), but there was no significant difference between SLE and control group in the periphery. Note the difference between central and peripheral densities both in controls and in patients with SLE (^∗∗^ *P* < 0.05 difference, Wilcoxon test).

We also compared the central cornea to the peripheral cornea in both controls and SLE patients (Wilcoxon test). The peripheral LC density was greater in the periphery than in the centre in SLE patients and in the control group (*P* < 0.001). LCM showed more than twofold higher values in the periphery than in the centre in controls and SLE (Table 2, *P* < 0.001).  Figure 3 summarizes central and peripheral LC densities in SLE patients and healthy volunteers.

## 4. Discussion

Ophthalmic manifestations of SLE have been widely discussed in the literature; however the majority of SLE patients do not develop ocular inflammatory symptoms throughout the course of their illness [25–27]. Ocular surface dendritic cells have been examined in many ophthalmic and systemic diseases including Sjögren's syndrome, RA, and AS [14, 19, 20]. As inflammation plays a role in ocular surface diseases, dendritic cells have been rediscovered as subjects of greater attention lately. In our study a marked increase of dendritic cells could be demonstrated by in vivo confocal corneal microscopy in the central cornea even in the absence of clinically manifested ocular inflammation and without overlapping Sjögren's syndrome. Additionally, more than half of these LCs showed an activated phenotype with small or long dendrites. There is no substantial evidence on correlation between LCs maturation and LC morphology; however it has been postulated that LCs with longer protrusions represent a more activated state, presumable with more cytokine production. The representation and distribution of LCs in SLE were similar to RA and AS [1, 20].

The results suggest that an increased antigen-presenting activity of LCs might operate in the cornea of SLE patients. Theoretically, it could be a consequence of an increased antigen supply or an intrinsically higher dendritic cell activity. In SLE, an enhanced interferon-*α* production of dendritic cells triggered by abnormal apoptotic material induces the hyperactivity of multiple effector pathways, and it is considered as a key pathogenic feature of the disease [28]. The signs of LC activation might be a consequence of continuous triggering by the immune complex deposits [28]; however in vivo confocal microscopy is not capable of demonstrating their presence in the cornea. It can be explained by the limitation of the magnification and resolution properties of the in vivo confocal microscope used in this study. These studies also demonstrated that recruitment and maturation of LCs can be induced by several proinflammatory cytokines including interleukin- (IL-) 1*α*, tumor necrosis factor- (TNF-) *α*, IL-6, IL-8, and IL-12 [29]. Both IL-1 inhibitors and anti-TNF-*α* therapy and corticosteroid therapy effectively suppressed the LC migration in vitro [29]. Indeed, deregulated cytokine production contributes to immune dysfunction and mediates tissue inflammation and organ damage in SLE. Inflammatory cytokines, like type I and type II interferons and IL-6, IL-1, and TNF-*α* as well as immunomodulatory cytokines like IL-10 and transforming growth factor *β* (TGF-*β*), have been identified as important players in SLE and are present in the tear of dry eye patients [30].

Langerhans cells belong to a group of dendritic cells that can manifest in different subtypes highlighting their activities. Zhivov et al. [13, 17] provided a confocal microscopic (in vivo) subtype definition which was further ameliorated and scored and applied in our present study similar to our previous works. Ex vivo, Hamrah et al. performed PCR, immunohistochemistry, and flow cytometry to further investigate LCs [8]. They have shown that Langerin (a c-type lectin expressed by specific dendritic cell populations which recognizes glycosylated patterns on pathogens) is specific for the LCs in corneal epithelium, whereas in corneal stroma different types of dendritic cells participate in antigen presentation.

Pathophysiological theories suggested and basic science research proved that, in aqueous deficiency states such as Sjögren's syndrome, reduced hydration of the ocular surface may initiate or contribute to the development of dry eye by increasing apoptosis and further induction of proinflammatory cytokines including IL-1, IL-6, IL-8, and TNF-*α* [30]. The accumulated cytokines have the capacity to decrease tear production via neuronal and hormonal effects [31]. Cordero-Coma et al. showed that, taking control of the underlying systemic inflammatory disease, tear production can improve which further supports our theory [32]. The possible higher concentration of cytokines in the tears of SLE patients might indirectly impair tear production and it might partly explain the dry eye mechanism without the presence of a true overlapping Sjögren's syndrome. Our results are in concordance with Villani's [33] data suggesting that the alterations of cornea are associated with the systemic inflammatory effect of the autoimmune disease.

The prevalence of sicca symptoms in autoimmune disease such as SLE has been reported as 36% in a study by Wangkaew et al. [25] and 28% by Gilboe et al. [22]. Our study demonstrated that ocular sicca symptoms are usually underdiagnosed in clinical practice. No patient had marked dry eye complaints, but significant changes in dry eye parameters could be identified in our study. A majority of our patients were females, which is in concordance with the gender-dependent nature of SLE. Previous studies found that SLE affects women more than men [34], and dry eye itself is more prevalent in women than in men [35], which shaped our intention to match the gender ratio of the control group.

It has been postulated that corneal immune status is interrelated with dysfunctional tear production, but the exact causative relationship is still not known in detail. The current study could not provide exact evidence on the relationship between LC density and dry eye parameters, but it could be demonstrated that both activated antigen-presenting cells and dry eye are detectable even in well controlled SLE patients. This lack of a direct correlation may partly be explained by the immune privileged status of the cornea. Another explanation could be that patients in our study were well controlled (were on complete or partial systemic immunosuppression) with low SLEDAI index, and we excluded patients with secondary Sjögren's syndrome.

Our results allow us to conclude that SLE alters LC density and morphology; it subsequently modifies corneal homeostasis and might contribute to the development of dry eye. Increased corneal LC density might be interpreted as an indicator of increased surveillance activity of the innate immune system. Our hypothesis that characterization of LC density and morphology in SLE patients could serve as a potential biomarker of disease activity in SLE patients with various disease severity needs further evidence to be proved. LC density alone could not be a descriptor of disease severity since SLE is a multifactorial disease and one parameter in itself probably cannot exactly describe all aspects of the disease. The autoimmune diseases and subsequent systemic therapies interfere with corneal homeostasis and undoubtedly play a role in LC maturation and kinetics. The limitation of our study is that our SLE patients were on systemic immunosuppressive treatment, resulting in controlled systemic inflammation. Investigation of therapy-naive SLE patients with more pronounced systemic inflammation would also be interesting.

Further investigations are required to clarify the role of adaptive immunity in the cornea in systemic inflammatory rheumatic diseases and the connection between the dry eye mechanism, the systemic inflammation, and the LCs.

## Figures and Tables

**Table 1:** Dry eye related parameters in different subgroups of SLE patients.

Study group	LIPCOF	Schirmer (mm/5 min)	TBUT [s]	OSDI
Healthy individuals	1.24 ± 0.54 (0–3)	11.67 ± 3.21 (6–16)	11.09 ± 3.37 (5–16)	11.06 ± 7.18 (0–35)
All SLE patients	1.36 ± 0.62 (0–3)	8.45 ± 9.82^*^ (2–40)	6.86 ± 3.53^*^ (2–15)	25.95 ± 17.92^*^ (4.5–7.4)
SLE patients according to SLEDAI score				
0 (29%)	1.37 ± 0.74 (0–2)	7.62 ± 7.34 (0.5–20)	5.12 ± 1.55^*^ (3–8)	16.82 ± 11.91 (4.54–35.71)
1–8 (60%)	1.29 ± 0.58 (1–3)	9.86 ± 11.11 (0.2–40)	8.23 ± 3.78^*^ (3–15)	29.64 ± 20.15^*^ (9.09–70.45)
>8 (11%)	1.66 ± 0.57 (1-2)	2.16 ± 0.76^*^ (1.5–3.0)	3.66 ± 1.52^*^ (2–5)	29.37 ± 11.06^*^ (16.6–36.1)
SLE patients according to CRP (mg/L)				
≤5 (33%)	1.57 ± 0.97 (0–3)	5.64 ± 5.08^*^ (1–15)	5.28 ± 2.49^*^ (2–10)	16.49 ± 10.52 (4.54–35.4)
>5 (67%)	1.28 ± 0.46 (1-2)	9.31 ± 10.68^*^ (0.2–40)	7.38 ± 3.72^*^ (3–15)	29.10 ± 18.93^*^ (5.55–70.45)

Data are expressed as mean ± SD. Comparisons between healthy individuals and SLE patients were made with Mann-Whitney tests. ^*^ *P* < 0.05. Subgroups were compared to each other and to healthy individuals with Kruskal-Wallis and Mann-Whitney tests (not found, thus not marked in table).

CRP: C-reactive protein; SLE: systemic lupus erythematosus; SLEDAI: SLE disease activity index; TBUT: tear break-up time; OSDI: ocular surface disease index.

**Table 2:** Confocal microscopy results in different subgroups of SLE patients.

Study group	LC centr. (cell/mm^2^)	LC periph. (cell/mm^2^)	LCM centr.	LCM periph.
Healthy individuals	20.57 ± 21.04 (0–71)	78.00 ± 39.51 (26–223)	1.00 ± 0.69 (0–3)	2.35 ± 0.54 (0–3)
All SLE patients	43.08 ± 48.67^*^ (1–169)	124.78 ± 165.39 (19–914)	1.43 ± 0.79^*^ (0–3)	2.89 ± 0.42^*^ (0–3)
SLE patients according to SLEDAI score				
0 (29%)	45.84 ± 43.1^*^ (1.8–125)	195.1 ± 292.9 (38.7–914)	1.63 ± 0.91 (1–3)	2.87 ± 0.35^*^ (2-3)
1–8 (60%)	41.26 ± 49.35 (2–169)	90.19 ± 64.76 (19–277)	1.41 ± 0.71^*^ (0–3)	2.88 ± 0.48^*^ (1–3)
>8 (11%)	46.10 ± 76.99^*^ (1–135)	133.20 ± 73.44 (48–179)	1.00 ± 1.00 (0–2)	3.00 ± 0.00^*^ (3-3)
SLE patients according to CRP (mg/L)				
≤5 (75%)	39.96 ± 50.20 (1.8–169)	135.6 ± 188.90 (19.8–914)	1.57 ± 0.81^*^ (0–3)	2.86 ± 0.48^*^ (1–3)
>5 (25%)	52.43 ± 46.03^*^ (1–135)	92.31 ± 50.09 (35–171)	1.00 ± 0.57 (0–2)	3.00 ± 0.00^*^ (3-3)
SLE patients according to Schirmer test (mm/5 min)				
<10 (61%)	42.03 ± 42.62 (1–135)	147.37 ± 204.04 (35–914)	1.58 ± 0.87 (0–3)	2.91 ± 0.24^*^ (2-3)
≥10 (39%)	44.68 ± 59.01^*^ (1.8–169)	89.85 ± 70.92^*^ (19.8–277.3)	1.18 ± 0.60^*^ (0–2)	2.81 ± 0.60^*^ (1–3)

Data are expressed as mean ± SD. Comparisons between healthy individuals and SLE patients were made with Mann-Whitney tests. ^*^ *P* < 0.05. Subgroups were compared to each other and to healthy individuals with Kruskal-Wallis and Mann-Whitney tests (not found, thus not marked in table).

CRP: C-reactive protein; SLE: systemic lupus erythematosus; SLEDAI: SLE disease activity index.
